# Targeting the Retinoic Acid Pathway to Eradicate Cancer Stem Cells

**DOI:** 10.3390/ijms24032373

**Published:** 2023-01-25

**Authors:** Geoffrey Brown

**Affiliations:** School of Biomedical Sciences, Institute of Clinical Sciences, College of Medical and Dental Sciences, University of Birmingham, Edgbaston, Birmingham B15 2TT, UK; g.brown@bham.ac.uk; Tel.: +44-(0)121-414-4082

**Keywords:** all-*trans* retinoic acid, retinoic acid receptor γ, oncogenes, cancer stem cells, carcinomas

## Abstract

All-*trans* retinoic acid is a morphogen during embryogenesis and a teratogen. Cancer is an error of development, and the retinoic acid receptors (RAR) for all-*trans* retinoic acid play a role in cancer. Expression of the cytosolic aldehyde dehydrogenases, which mediate the last step to the synthesis of all-*trans* retinoic acid, is deregulated in various human cancers. Inhibiting these enzymes using a variety of agents reduced the proliferation of lung cancer cells, reduced the proliferation and induced apoptosis of ovarian, prostate, squamous, and uterine cancer cells, and sensitised breast, colorectal and ovarian cancer cells to chemotherapeutic agents. RARγ is an oncogene within some cases of AML, cholangiocarcinoma, colorectal cancer, clear cell renal cell carcinoma, hepatocellular carcinoma, pancreatic ductal adenocarcinoma, prostate cancer, and ovarian cancer. Pan-RAR and RARγ antagonist inhibition of the action of RARγ led to necroptosis of human prostate and pediatric brain tumour cancer stem cells. Treatment of hepatocellular carcinoma cells with the flavenoid acacetin, which interferes with the action of RARγ, decreased cell growth and induced apoptosis. Targeting the retinoic acid pathway is promising regarding the development of new drugs to eradicate cancer stem cells.

## 1. Introduction

All-trans retinoic acid (ATRA) plays a crucial role in the embryonic development of the forebrain, hindbrain, forelimb buds, heart, lung, and skeleton, as revealed by the defects that are provoked by vitamin A deficiency within chicken and mammalian embryos [1]. For the proper conduct of stem cell differentiation, the levels of ATRA are tightly controlled across the embryo by enzymes and cells that synthesise and degrade, creating distinct gradients and boundaries [2]. Disruption of this complex process to the right level of ATRA has a profound biological consequence, as retinoids are teratogenic. The application of exogenous ATRA to rodent embryos led to anterior/posterior axis malformations and to the structures arising from the axis [3].

ATRA regulates cell development by controlling gene expression via activation of the three main isoforms of the closely related retinoic acid receptors (RARs) α, β, and γ. They bind as a heterodimer with retinoid X receptor to the cis-acting response elements of ATRA target genes to allow gene transcription when ATRA binds to RARs [4]. The response elements are associated with a substantial number of genes. When ATRA is absent, RAR/RXR heterodimers bound to DNA are associated with corepressors which, having recruited histone deacetylase complexes, maintain chromatin in a condensed state and gene promoters in a repressed state. Cancer has been described as “a developmental disturbance or an error of development” [5], and the crucial roles of RARs are emphasised by their diverse actions in the treatment and prevention of cancer. The use of ATRA has revolutionised the treatment of acute promyelocytic leukaemia [6], and, for example, the RARβ2 splice variant is a well-known tumour suppressor which is silenced epigenetically in human cancers [7].

The concept of cancer stem cells (CSCs) is central to an understanding of the development and nature of cancer [8]. They are thought to arise largely from the transformation of a tissue-specific stem or progenitor cell and, like normal stem cells, are at the apex of the cancer cellular hierarchy. Some CSCs are quiescent and spared by conventional chemotherapy that targets rapidly dividing cancer cells. For example, chronic myeloid leukemia arises in a hematopoietic stem cell, and the leukemia stem cells are resistant to the tyrosine kinase inhibitors that are used to treat this disease [9]. Accordingly, the leukaemia stem cells provide a source of cells for disease relapse and CSCs are thought to be responsible for carcinoma metastasis. For over a decade, the eradication of CSCs has provided a substantial challenge to the successful treatment of many cancers [10]. As for normal stem cell development, the supply of ATRA and the activation status of RARs are important to the behaviour of CSCs. This review examines perturbations to the uptake, storage, and metabolism of retinoids and particularly the action of RARγ within cancer cells. Consideration is given to various ways of targeting the retinoic acid pathway as an avenue to eradicate CSCs.

## 2. The Metabolism of Dietary Retinol to ATRA

**Figure 1:**
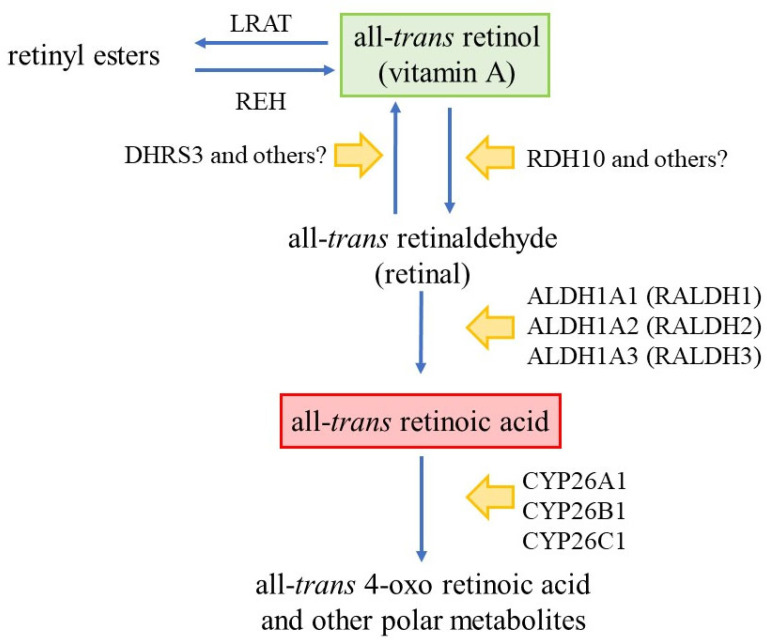
The metabolism of all-*trans*-retinol into ATRA. All-*trans*-retinol is mainly taken up into cells via the retinol-binding protein 4 (RB4) blood transport protein binding to the specific cell surface receptor Stra6. LRAT, lecithin retinol acyltransferase; REH, retinyl ester hydrolase; DHRS3, retinaldehyde reductase 3; RDH10, retinol dehydrogenase 10; ALDH, aldehyde dehydrogenases; CYP, cytochrome P450.

Numerous proteins and a complex series of events are required to supply cells with ATRA (Figure 1; reviewed in [11,12]). Virtually allthe dietary all-*trans*-retinol (vitamin A) is transported in the bloodstream by the retinol-binding protein 4 (RBP4), and the transfer from RBP4 into cells is mediated by binding of RBP4 to the specific cell surface receptor stimulated by retinoic acid 6 (Stra6) [13]. Cells take up retinyl esters transported by either chylomicrons or lipoproteins.

Within cells, all-*trans*-retinol may be isomerised into 9-*cis*- or 11-*cis*-retinol. In the cytoplasm, the cellular retinol-binding protein type I (CRBPI) sequesters all-*trans*-retinol in a high-affinity complex and delivers it to the endoplasmic reticulum membranes for either esterification, by lecithin retinol acyltransferase (LRAT), to retinyl esters for storage, or dehydrogenation into all-*trans*-retinaldehyde (retinal). This first oxidative step is rate-limiting and reversible, and there is ambiguity regarding the integral membrane enzymes that are physiologically relevant. Oxidation is controlled by retinol dehydrogenase 10 (RDH10) and possibly other dehydrogenases of the short-chain alcohol dehydrogenase/reductase gene family [14]. Retinaldehyde reductase 3 (DHRS3), and possibly other reductases, reduce all-*trans*-retinaldehyde back to all-*trans*-retinol. All-*trans*-retinaldehyde is oxidised to ATRA, and there is a consensus regarding the enzymes. This step is irreversible and catalysed by the cytosolic aldehyde dehydrogenases (ALDH) ALDH1A1 (RALDH1), ALDH1A2 (RALDH2), and ALDH1A3 (RALDH3) [11]. ATRA binds to the two high affinity and homologous proteins cellular retinoic acid binding-protein I (CRABP-I) and cellular retinoic acid binding protein II (CRABP-II). They deliver ATRA to the enzymes that are responsible for its destruction (via CRABP-I) or to the nucleus and the RARs that mediate ATRA’s biological action (via CRABP-II) [15]. ATRA is catabolised to polar metabolites for elimination by the body by the cytochrome P450 26 (CYP26) enzymes CYP26A1, CYP26B1, and CYP26C1 [16,17].

## 3. The Biological Actions of ATRA within Normal Cells

ATRA is the key biologically active metabolite of vitamin A, and activated RARα, RARβ, and RARγ have been described as the conductors of the retinoic acid symphony during development [18]. Expression of RARα, RARβ, and RARγ changes as hematopoietic stem cells and organisms develop. RARγ is selectively expressed by hematopoietic stem cells and primitive progenitors. As these cells mature, a fall in the expression of RARγ was mirrored by an increase in the expression of RARα and RARβ [19]. RARs α, β, and γ paralogs are expressed ubiquitously with zebrafish embryos at 8 h post fertilisation. RARγ transcripts were restricted to primitive cells at later stages of embryo development. At 24 h post fertilisation, they were restricted to mesodermal and neural crest stem and progenitor cells, in the head area, in the lateral plate mesoderm, and in the pre-somitic mesoderm of the tail bud, and RARγ expression was still visible in the tail bud at 48 h post fertilisation [20]. From the analysis of the consequences of RAR subtype-specific knockdown to zebrafish development, RARγ plays a role in the basal regulation of ATRA-target genes, whereas RARα regulates target genes upon exposure to exogenous ATRA. The subtypes appear to discriminate genes and drive transcription in a subtype-specific manner [21].

A longstanding finding is that ATRA enhances the neutrophil differentiation of late myeloid progenitors from murine bone marrow [22] and drives promyeloid cell lines, such as HL60, to differentiate towards neutrophils [23]. Pharmacological and genetic studies have revealed that RARα is a bifunctional regulator of neutrophil differentiation whereby RARα is a negative regulator when not complexed with ATRA and promotes differentiation when ATRA or a specific RARα agonist is bound [24,25]. By contrast, RARγ is a critical regulator of HSC maintenance because the number of HSCs was much reduced in bone marrow cells from RARγ knockout mice, and there was an increase in the number of more mature progenitors [19].

Regarding hematopoietic stem cell maintenance, active RARγ may also block stem cell differentiation. This is the case for zebrafish embryos because the development of tissues from the cranial neural crest and primitive mesoderm stem cells was dependent on a lack of activation of RARγ. Treatment of zebrafish embryos at 3 days post fertilisation with a RARγ-selective agonist blocked stem cell development to prevent fin, bone, and neural ganglia formation. The agonist did not substantially affect stem cell presence as fin formation was restored by the addition of a RARγ antagonist to reverse the action of the RARγ agonist or wash out of the RARγ agonist [26]. The activity of RARγ is important to murine embryonic stem cells because knockout studies showed that RARγ function was required for the expression of ATRA-regulated transcripts and that a functional RARγ was essential for chromatin remodelling and DNA epigenetic marks [27].

**Figure 2:**
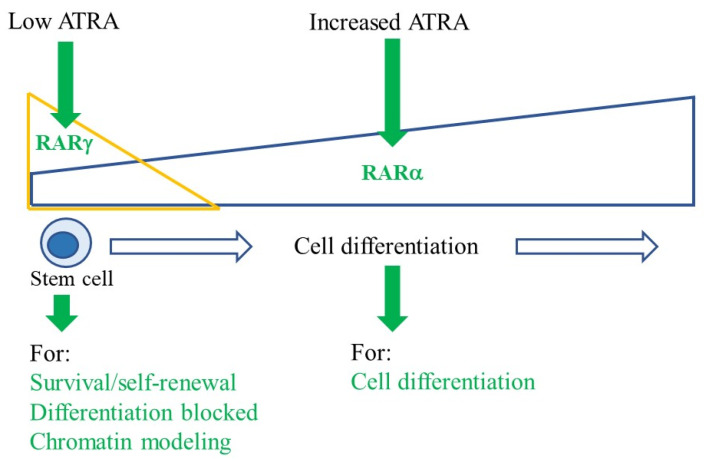
Active RARγ and RARα control stem cell maintenance and differentiation, respectively RARγ expression is restricted to stem cells and their immediate offspring, and as these cells differentiate, a fall in the expression of RARγ is mirrored by an increase in the expression of RARα. RARγ functions to maintain stem cells when ATRA is absent or low. ATRA activation of RARα promotes cell differentiation.

Therefore, RARγ and RARα play reciprocal roles in the conduct of stem cell behaviour (Figure 2). The activity of RARγ is essential to stem cell maintenance and blocks differentiation. RARγ is important to the basal level of gene expression when ATRA is low or absent and to chromatin modelling and DNA epigenetic marks. By contrast, ATRA activation of RARα promotes differentiation.

## 4. Defective Retinoid Metabolism within Cancer Cells

The cellular machinery that is involved in all-*trans*-retinol uptake and metabolism is often perturbed in cancer cells suggesting that a change to the availability of ATRA and signalling is important to cancer development and the behaviour of CSCs [28]. The retinoid machinery is subverted or co-opted by cancer cells in various ways. Expression of the aldehyde ALDH1 family of enzymes, which oxidise all-*trans*-retinaldehyde to ATRA, is critical to the cell synthesis of ATRA with an absence or overexpression within cancer cells leading to ATRA insufficiency or excess, respectively. For some cancer cells, the uptake of all-trans-retinol is perturbed by increased expression of Stra6. Retinyl esters store all-*trans*-retinol within cells, and perturbation to this conversion within some cancer cells leads to ATRA insufficiency. Finally, the delivery of ATRA to the nucleus and RARs is abnormal within cancer cells that have a high level of CRABPII.

Stra6 (for all-*trans*-retinol uptake) mRNA was upregulated in human breast cancer as compared to normal breast tissue and elevated in colorectal cancer, and undetectable in normal colon tissue. HCT116 human colorectal cancer cells that stably over-expressed Stra6 developed substantially larger tumours than parental cells in athymic nude mice. Stra6 over-expression also promoted the oncogenic potential of MCF-7 breast cancer cells. Stra6 was critical for tumour formation by colorectal cancer cells because of stable down-regulation within the human primary adenocarcinoma SW480 cells by means of an shRNA vector, markedly suppressed tumour formation in athymic nude mice [29]. The oncogenic activity of Stra6 was mediated by STAT3, which is a known driver of cancer [30]. A high-fat diet is associated with ~80% of cases of colorectal cancer, and the Stra6-RBP4 pathway plays a role because a high-fat diet increases the level of Stra6 [31].

Defects in the esterification of all-*trans*-retinol to retinyl ester (by LRAT) interfere with the storage and metabolism of retinol, leading to a local ATRA deficiency. Whilst the basal levels of mRNA for Stra6 were the same for the human prostate cancer cell line PC-3 and normal prostate epithelium cells, the level of LRAT mRNA in PC-3 cells was lower than that in normal prostate epithelium cells [32]. Other human cancers, including cancers of the bladder, breast, kidney, oral cavity and skin, had decreased LRAT expression and abnormally low intracellular retinyl ester levels [33]. CRBP1 is also required for the conversion of all-*trans*-retinol to retinyl ester, and the loss of CRBP1 expression and gene promoter methylation has been commonly observed for patients’ prostate cancer samples [34].

As mentioned above, the aldehyde ALDH1 family, which includes ALDH1A1, ALDH1A2, and ALDH1A3, oxidises all-*trans*-retinaldehyde to ATRA. Aldehyde dehydrogenase activity was not detected in an extract from a patient’s prostate cancer, whereas activity was readily detected in extracts from control normal prostate tissue and prostates with benign hyperplasia [35]. For the human prostate cancer cell line LNCaP, mRNAs for ALDH1A1 and ALDH1A2 were not detected: ALDH1A3 mRNA was present, and expression was androgen responsive [36]. Histological studies have compared the expression of ALDH1s in primary prostate cancer and normal prostate epithelium and confirmed a lack of expression of ALDH1A2 in primary prostate cancer. The *ALDH1A2* gene promoter was significantly hypermethylated in primary prostate cancer as compared with normal prostate epithelium, which expressed ALDH1A2 [37]. By contrast to prostate cancer, various cancer types overexpressed ALDH1A1, including breast, colorectal, oesophagus, liver, lung, ovary, pancreas, and stomach. The influence of overexpression on prognosis varies for different cancers from favourable to poor, and to no difference (reviewed in [38]). A low level of expression of ALDH1A2 has been associated with a poor prognosis and shorter disease-free and overall survival for ovarian cancer patients. From complementary DNA microarray data, ALDH1A1 was downregulated in human ovarian cancer cell line cells, and hypermethylation of *ALDH1A2* was higher in the cancer cell lines than in immortalised human ovarian epithelial cell lines [39]. A low level of expression of ALDH1A2 in head and neck squamous cell carcinoma patients’ cells has been related to an unfavourable prognosis and a mesenchymal-like phenotype leading to the suggestion that patients might benefit from treatment with retinoids [40]. The predominant expression of ALDH1A3 within patients’ glioblastomas was associated with shorter overall survival [41], and protein expression correlated positively with the stage for triple-negative breast cancer samples [42].

The expression of CRABPII, which transports ATRA into the nucleus, is abnormal within breast, hepatocellular, and lung cancers. CRABPII expression was higher in estrogen receptor-positive breast cancer tissues than in surrounding tissue, and there was faint staining of estrogen receptor-negative breast tissue. Knockdown of expression within the human estrogen receptor-positive T47D and MCF7 cell lines promoted epithelial-mesenchymal transition and metastasis in vitro and in vivo. The reverse was the case for the estrogen receptor-negative cell lines BT549 and MDA-MB231, whereby overexpression promoted epithelial-mesenchymal transition and metastasis in vitro and in vivo [43]. Expression levels were elevated in hepatocellular carcinoma tissue and cell lines when compared with normal human L-02 liver cells. Knockdown of CRABPII expression, by shRNA, within the human cell line HepG2 inhibited the migration, invasion, and colony formation of cells and tumorigenesis and angiogenesis in vivo [44]. Analysis of clinical samples of lung cancer revealed an association between a high level of CRABPII expression and lymph node metastasis, poor overall survival, and increased recurrence. CRABPII was expressed at a high level in a high-metastatic subline of C10F4 lung cancer cells as compared to low-metastatic lung cancer cells, and CRABPII knockdown decreased migration, invasion, and in vivo metastasis [45].

There are various perturbations to the uptake of all-trans-retinol and metabolism to ATRA within cancer cells, but there isn’t a profile to the supply of ATRA that typifies cancer cells. The uptake and storage of all-*trans*-retinol are sometimes reduced. The extent to which there is metabolism to ATRA is confused by expression of ALDHs in cancer versus normal cells varies from up-regulation to down-regulation and whether such does or does not relate to a poor prognosis. Regarding down-regulation, ALDHs have been proposed as a tumour suppressor. However, a reduced level of ALDH expression and ATRA production does not necessarily infer a tumour-suppressive role for the gene because RARγ is exquisitely sensitive to ATRA transactivation and an oncogene for some cancers (see below). Findings for up-regulation versus down-regulation of ALDHs may be confounded by only a few of the 19 different ALDH isoforms that have been shown to play a role in the ATRA pathway, and other isoforms may be important. Whether the paired cancer versus normal cells investigated have differentiated to the same degree is also important because the levels of expression of ALDH1A1, ALDH1A2 and ALDH1A3 change during development, and, for example, parallel rat nephron development [46]. The delivery of ATRA to the nucleus of cancer cells seems to be ensured because CRABPII is present or over-expressed.

## 5. Some Cancer Cells Reside in a Low ATRA Environment

The cellular machinery that is required to generate ATRA is clearly deficient in malignant prostate cancer because the intracellular level of ATRA for patients’ prostate cancer tumours is at or near the limit of detection of ~1 ng/g tissue. Adjacent normal prostate epithelium cells and benign hyperplasia cells see ~8 times more ATRA [47]. For prostate cancer, and as above, this could reflect a reduced/lack of activity of ALDH1A1 and ALDH1A2, which oxidise all-*trans*-retinaldehyde to ATRA. Another possible cause is that there is a more rapid degradation of ATRA to 4-oxo-RA and other polar RA metabolites. Prostate cancer tumours contain increased amounts of cellular retinoic acid-binding protein [48,49]. Increased CRABP-1 may have accelerated ATRA degradation, as shown for F9 teratocarcinoma cells, whereby increased expression influenced the amounts and types of ATRA metabolites [50].

**Figure 3:**
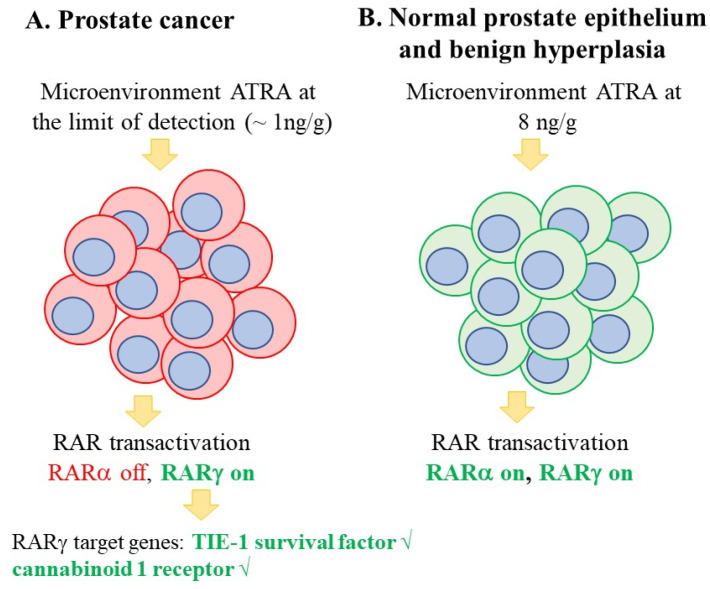
Patients’ prostate cancer cells reside in a low ATRA environment. For tumours, the intracellular level of ATRA is at or near the limit of detection (at ~1 ng/g tissue), and adjacent normal prostate epithelium cells and benign hyperplasia cells see ~8 times more. RARγ is activated at 0.24 nM ATRA and RARα at 19.3 nM. Evidence of RARγ usage within prostate cancer cells is that TIE-1 and the cannabinoid 1 receptor are overexpressed as shown by √.

Most primary prostate cancer cells and the prostate cancer cell lines DU-145 and PC-3 express RARα and RARγ; the LNCaP cell line additionally expresses RARβ. ATRA binds to each of the RAR isoforms with a similar affinity (typical K_d_’s 5–20 nM). The low intracellular level of ATRA in prostate cancer tumours is important because the RAR isoforms have a differential sensitivity towards ATRA transactivation: RARγ is activated at a much lower concentration of ATRA than RARα. A sub nM level of ATRA (0.24 nM) was sufficient to transactivate RARγ within LNCaP cells, whereas a level of 19.3 nM was needed to activate RARα [51]. RARγ-driven gene expression is favoured within prostate cancer cells due to their low intracellular level of ATRA (Figure 3) because there is differential RARγ usage in prostate cancer tumours as revealed by the target genes are over-expressed. They include the genes that encode the pro-angiogenic/survival factor TIE-1, as seen in a transgenic mouse model of prostate adenocarcinoma [52], and the cannabinoid 1 receptor, whereby a high expression in patients’ prostate cancers is associated with disease severity and a poorer outcome [53].

A low ATRA environment may be generated by the environment that cells reside in. Expression of the retinoid metabolising CYP26 within the microenvironment has been shown to influence the fate of normal hematopoietic stem cells by protecting these cells from ATRA-induced differentiation and promoting their self-renewal [54]. The ATRA-induced differentiation of acute promyelocytic leukemia cells and non-acute promyelocytic leukemia cells was blocked by bone marrow stromal cells. This was attributed to the bone marrow stromal cells producing a precipitous drop in the local level of ATRA, and cell sensitivity to ATRA was restored by inhibiting CYP26. The postulate from these findings was that AML cells reside in a low ATRA sanctuary that is created by bone marrow stromal cells, which also protect AML cells from ATRA therapy [55]. Patients’ breast CSCs may also reside in a low ATRA sanctuary because putative CSCs from cell lines isolated from mammary tumours from mammary tumour virus LTR-polyoma middle T antigen transgenic mice and which generated highly aggressive tumours in NOD SCID mice did not express the enzymes to metabolise retinol into ATRA [56]. Similarly, none of the malignant ovarian cancer cell lines examined produced a detectable amount of ATRA, which was attributed to a complete loss of ALDH1A2, and this loss was linked to tumour growth. However, ATRA, which is lipophilic, passes freely between cells and normal ovarian surface epithelial cells expressed ALDH1A2 and produced ATRA, which may be made available to the patients’ tumour cells [57].

**Figure 4:**
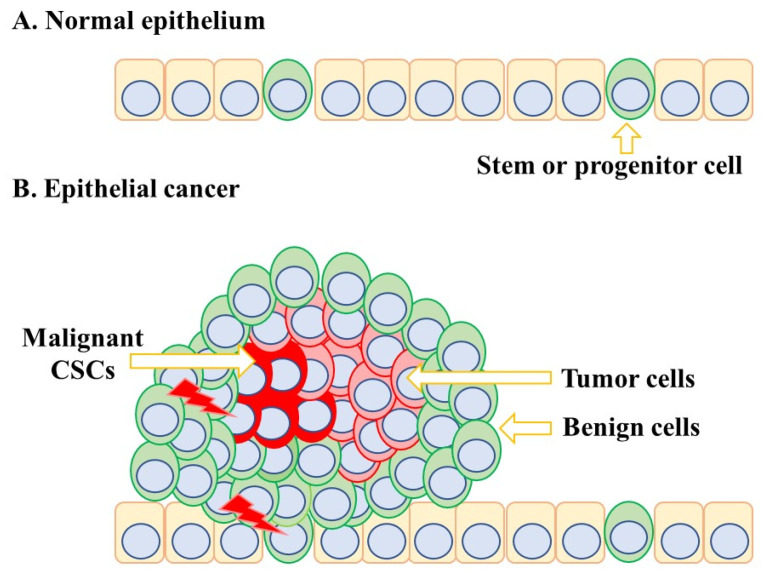
Normal epithelial stem cells and carcinoma stem cells reside in different environments. Normal epithelial stem cells are scattered and intermittent throughout the normal epithelium. A proposal regarding epithelial carcinoma is that a first oncogenic insult leads to a benign and homogeneous population of cells, with a second oncogenic insult giving rise to a malignant and invasive clone. CSCs overtake the tumour after a final hit.

We do not know for certain the extent to which CSCs can synthesise ATRA nor where cancers stem cells reside within a tumour and whether ATRA is provided by adjacent cells. The complex architecture of solid tumours is an important consideration in the supply of ATRA to CSCs because their environment is very different from that of a normal tissue-specific stem cell (Figure 4). The stem or progenitor cells for normal epithelium are scattered and intermittent throughout the epithelium. The architecture of epithelial carcinomas such as pancreatic cancer is a landscape of subclones due to the evolution of mutations [58]. A theoretical proposal for epithelial carcinomas is that a first oncogenic insult leads to a benign and homogeneous population of cells. A malignant and invasive clone arises from a second oncogenic insult. A further hit to the malignant subclone leads to further transformation, and subclones that are genetically different coexist within the tumour. CSCs overtake the tumour after a final hit, and for some cancers, the frequency of CSCs is high. For example, around 27% of unselected melanoma cells from four patients were able to give rise to tumours in a xenotransplantation assay [59]. Leukaemia stem cells are viewed as rare cells, but these cells can create their own environment. Studies of a mouse xenograft model of human Nalm-6 pre-B acute ALL cells have shown that they create abnormal bone marrow vascular niches [60], which may compromise the supply of ATRA.

## 6. RARγ Is an Oncogene for Various Cancers

RARγ plays a role in the pathophysiology of the prostate because it is abnormal in RARγ knockout mice. Squamous epithelium had replaced glandular epithelium resulting in the absence of the typical prostatic secretion products [61]. This transformation from a glandular to a squamous epithelium was also seen in much earlier studies of the prostates of mice that were fed a vitamin A-deficient diet [62]. As mentioned above, patients’ prostate cancer cells appear to have adapted to survive and grow in an abnormally low ATRA environment and may then be reliant on RARγ activation for survival and growth. RARγ activation is important to prostate cancer cell proliferation because the use of a RARγ agonist or a low level of ATRA (0.1 to 1 nM) to activate just RARγ stimulated the growth of and colony formation by prostate cancer cell line cells [63]. These findings support the view that RARγ is an oncogene for prostate cancer.

In the SWOG9157 phase II trial of oral ATRA in hepatocellular carcinoma, the clinical impression was that the patients progressed rapidly, suggesting that ATRA had promoted disease [64]. RARγ is an oncogenic protein for hepatocellular carcinoma because it is significantly elevated in most human tumour tissue samples and the human hepatocellular carcinoma HepG2, QCy-7703, and QSG-7701 cell line cells. Over-expression of RARγ promoted colony formation by hepatocellular carcinoma cells in vitro and the growth of xenografts, and the use of siRNA to down-regulate expression impaired ATRA induction of the disease marker alpha-fetoprotein [65].

A growth-stimulatory response to ATRA was seen in a patient with relapsed acute myeloid leukemia. The patient died eight days post continuous ATRA treatment from very rapid disease progression. An increase in the level of nuclear RARγ was seen in the patients’ primary cells when treated in vitro with ATRA, and the cells proliferated rapidly in response to a RARγ agonist [66]. The association between rearrangements that involve the gene that encodes RARα and the pathophysiology of acute promyelocytic leukaemia is well established, and recently, gene rearrangements that involve the gene that encodes RARγ have been described for nine acute promyelocytic leukaemia patients. Fusions were observed between the gene that encodes RARγ and the genes for NUP98 (three patients), CPSF6 (four patients), NPM1 (one patient) and PML (one patient). Other than the patient with PML translocation, the patients failed to respond to ATRA-based treatment [67]. Hence, *RARγ* is an oncogene for some AML patients.

RARγ is also an oncogene for human cholangiocarcinoma, clear cell renal cell carcinoma, colorectal cancer, and pancreatic ductal adenocarcinoma. RARγ over-expression in cholangiocarcinoma is associated with resistance to 5-fluorouracil and a poor prognosis. For the human cholangiocarcinoma cell lines QBC939, SK-ChA-1, and MZ-ChA-1, siRNA knockdown of RARγ expression suppressed cell proliferation, and xenograft tumour growth in nude mice was reduced for stably transfected QBC939 cells [68]. Around half of the patients with clear cell renal cell carcinoma were observed to over-express RARγ, as seen from qPCR and a bioinformatics analysis [69]. RARγ mRNA and protein were frequently overexpressed in human colorectal cancer tissue as compared to non-tumourous colorectal tissue, and expression was increased in the cell lines HT29, HCT116, RKO and SW480 as compared to the normal colonic epithelial cells HCoEpiC. Knockdown of RARγ within the cell lines decreased expression of the multidrug resistance 1 protein and increased the sensitivity of the cell lines to 5-fluorouracil, oxaliplatin, and vincristine [70]. The levels of RARγ transcripts were significantly higher in pancreatic ductal adenocarcinoma tissue and high-grade precancerous lesions than in normal pancreatic tissue. Blocking of RARγ signalling via siRNA suppressed the cell proliferation of the pancreatic ductal adenocarcinoma cell lines PK-1 and Panc-1, arresting cells in G1 of the cell cycle without causing apoptosis. Treatment of five lumen-forming patient-derived pancreatic ductal adenocarcinoma organoids with siRNA revealed that RARγ signalling underlies their proliferation by promoting cell cycle progression [71].

RARγ plays an important role in ovarian cancer cell proliferation [72]. Patients with a high level of expression had a poor prognosis with high-level expression relating to FIGO stages III–IV and a survival time of <5 years. Accordingly, RARγ can be used as a diagnostic and prognostic biomarker. In in vitro experiments, the proliferation and colony formation capacity of ovarian cancer A2780 and SKOV3 cell line cells was suppressed by down-regulation of RARγ expression. Tumour cell growth by A2780 cells in nude mice was reduced by the knockdown of RARγ. For the ovarian cancer cell line cells, a high level of RARγ expression regulates cell proliferation because knockdown reduces the level of Ki-67 and the proliferating cell nuclear antigen. From GO enrichment of RARγ-related genes, the investigators concluded that RARγ influences gene regulation to enhance the proliferation and progression of ovarian cancer cells.

## 7. Inhibiting the Activity of ALDHs

**Figure 5:**
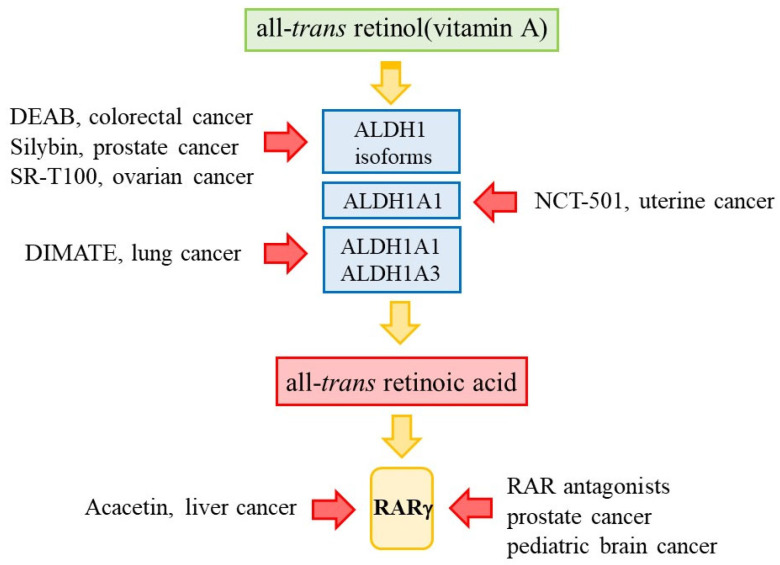
Targeting the retinoic acid pathway. Preventing the action of RARγ is druggable by either targeting the enzymes that generate a supply of ATRA or by blocking the action of RARγ.

That all-*trans*-retinol metabolism is deregulated within cancer cells and that RARγ is an oncogene within various cancers provide good support for targeting the retinoic acid pathway to eradicate cancer cells. The need is to prevent the action of RARγ by either targeting the enzymes that provide a supply of ATRA or by blocking the action of RARγ, and both are drugable (Figure 5).

Aside from the uncertainty regarding the upregulation or downregulation of ALDHs, the rationale for targeting to eradicate CSCs is that expression marks the clonogenicity and metastatic capacity of CSCs in colorectal cancer [73] and esophageal squamous cell carcinoma [74]. ALDH1A1 expression maintains the properties of squamous cell carcinoma CSCs by enhancing clonogenicity, the ability to generate spheroids in vitro, and tumorigenicity in vivo [75]. High ALDH activity identifies prostate cancer CSCs that are metastasis-initiating [76]. For non-small cell lung cancer primary cells and cell lines, high ALDH activity correlates with a poor prognosis. Notch signalling maintains ALDH+ lung cancer cells and suppression within non-small cell lung cancer cell lines by the use of a γ-secretase inhibitor, leading to reductions in ALDH+ lung cancer cells, tumour cell proliferation, and clonogenicity [77]. ALDH1A2 and ALDH1A3 have been proposed as markers of the CSC that initiates human neuroblastoma [78].

Silybin (HY-13748) is a mixture of flavonolignan and flavonoid polyphenolic compounds extracted from milk thistle seeds. The compounds have anticancer properties, and silybin suppressed the proliferation, migration, and invasive capacity of prostate cancer DU145 cell line cells in in vitro experiments. Silybin also reduced the volume and weight of tumours when prostate cancer cells were implanted into nude mice. These actions were attributed to the downregulation of ALDH1 mRNA and protein expression and inhibition of the activation of RARα [79]. There is gene amplification of *ALDH1A1*, *ALDH1A3*, or *ALDH3A1* or upregulation of mRNA in 31% of non-small cell lung cancers. DIMATE, an irreversible inhibitor of ALDH1A1 and ALDH1A3, was cytotoxic for non-small cell lung cancer cell lines and effective against orthotopic xenografts and enhanced cisplatin chemotherapy. Hydroxynonenal-protein adduct accumulation, the glutathione S-transferase omega 1-mediated depletion of glutathione, and increased H_2_O_2_ led to cell death in orthotopic xenografts [80]. ALDH1A1 expression has been associated with poor differentiation of human colorectal cancer cells and poor patient survival [73]. DEAB inhibition of ALDH1 isoforms sensitised the colorectal cancer cell lines HT-29/eGFP, HCT-116/eGFP, and LS-180/eGFP to chemotherapy. Inhibition of expression of ALD1A1 or ALDH1A3 by siRNA sensitised these cells to capecitabine and 5-FU and inhibited the proliferation of HT-29/eGFP cells in a subcutaneous xenograft model [81].

A high level of expression of ALDH1 isoforms has been associated with disease relapse of ovarian cancer [82]. An extract of the species of nightshade *Solanum inconum* (SR-T100) contains mainly the alkaloid solomargine, which induces apoptosis. Solomargine was more effective in decreasing the cell viability of ovarian cancer ES2, TOV-21G, IGROV1, and A2780 cell line cells than that of the non-malignant IOSE-398 cells, increased the sensitivity of ovarian cancer cell line cells to cisplatin and paclitaxel, and a combination of solomargine and cisplatin was effective in inhibiting the growth of the cell line A278OCP70 in mouse xenografts [83]. For A2780CP70 cells, solomargine downregulated the expression of ALDH1 isoforms, which mediate their stemness [83], and ALDH inhibitors were effective in eliminating ovarian cancer CSCs (reviewed in [84]). Solomargine induced the apoptosis of human breast cancer HDL-100, ZR-75-1, and SK-BR-3 cell line cells and sensitised these cells to cisplatin [85]. It induced apoptosis of the human cutaneous squamous cell carcinoma A431, SCC4, SCC9, and SCC25 cell line cells and suppressed their growth in mouse xenografts [86].

NCT-501 was identified as a specific inhibitor of ALDH1A1 from a systematic medicinal chemistry optimisation of theophylline-based compounds [87]. It reduced spheroid cell formation by patients’ uterine endometrial CSCs and caused selective cell death in cells with a high level of activity of ALDH [88]. 673A, an analogue of DEAB, is an inhibitor of ALDH1A isoforms. This agent-induced necroptosis of ovarian cancer CD133+ CSCs synergised with chemotherapy to reduce tumour initiation and promote tumour eradication in vivo [89]. Two compounds termed 13g and 13h, obtained from studies of the co-crystal structure of CM039 with ALDH1A1, were selective towards ALDH1A1 and, as to their efficacy in vivo against ovarian CSCs, provided potential adjuncts to chemotherapy. 13h synergised with cisplatin when tested against patient-derived spheroids [90].

The use of general and isoform-specific ALDH inhibitors to treat various cancers holds promise. Isoform-specific inhibitors of ALDHs are likely to be needed, and there is a need to develop inhibitors with improved pharmacokinetics. Expression of ALDHs in cells has also been blocked by inhibiting the action of regulators of ALDHs, including blocking the action of, for example, the transcriptional and epigenetic regulator bromodomain-containing protein 4 and the membrane protein regulator epithelial membrane protein. The various approaches to targeting ALDHs were effective in eliminating CSCs in gynecologic malignancies (reviewed in [84]).

## 8. Targeting the Action RARγ

It is clear that antagonising RARs is effective against CSCs. The pan-RAR antagonist AGN194310 and the RARγ antagonist AGN205728 were highly effective in driving growth arrest in G_1_ of cell cycle and death of primary cultures of patients’ prostate cancer cells and the human DU-145, LNCaP, and PC-3 cell line cells (IC_50_ values of 3.5 to 6 × 10^−7^ M). These lines were derived from metastatic disease. Though RARγ expression within cells is normally restricted to primitive cells, all the flask-cultured cells’ growth was arrested, and they died quickly. Hence, RARγ is expressed by most of the prostate cancer cells, suggesting that expression is deregulated within prostate cancer cells. CSC-like cells gave rise to large colonies when the prostate cancer cell line cells were dispersed in a petri dish, and both antagonists eliminated colony formation (IC_50_ values 16–34 × 10^−9^ M) [63,91,92]. The pan-RAR antagonist was also highly effective against primary cultures of two pediatric primitive neuroectodermal tumours and a pediatric astrocytoma. The cells that give rise to neurospheres are CSCs, and neurosphere formation was ablated [93].

The prostate cancer cells died via necroptosis, which was mitochondria depolarisation-dependent, involved cellular DNA fragmentation, and was caspase-independent [63,91]. Similarly, retinoid-deprived Jurkat T leukaemia cells died by necroptosis and via activation of the poly(ADP-ribose) polymerase PARP-1. PARP1 ribosylates various proteins to change their function, and the proteins include those involved in transcription and the cell cycle [94]. PARP-1 plays a central role in the cellular stress response to process diverse signals, and its activation leads to mitochondrial dysfunction and the release of ATP, NAD^+^ and the caspase-independent nuclease AIF, which fragments DNA [95]. Treatment of the prostate cancer cell lines with the PARP-1 inhibitor 1,5-dihydroisoquinoline blocked RAR antagonist provoked necroptosis of prostate cancer cell line cells.

RARγ is present in both the nucleus and cytoplasm of cells, and the natural flavonoid acacetin (5,7-dihydroxy-4-methoxyflavone) from *Flos Chrysanthemi indici* targeted the cytoplasmic action of RARγ within hepatocellular carcinoma cells. Acacetin has anticancer activity against various cancers, including lung, breast and prostate cancer, and strongly inhibits the growth and induced apoptosis of the human liver cancer HepG2, QGY-7703 and SMMC7721 cell line cells. From studies of hepatocellular carcinoma cells, investigators argued that the anticancer action of acacetin does not depend on the modulation of RARγ-driven gene transcription and instead that RARγ controls the balance between AKT- and p53-mediated events. The binding of acacetin to RARγ prevented its activation of AKT, and the recovery of normal p53 signalling followed. Acacetin targeting of cytoplasmic RARγ had, therefore, switched AKT-p53 from a pro-survival to a pro-apoptotic program [96]. Regarding intracellular signalling, RARγ also interacts with the p85α regulatory subunit of phosphoinositide 3-kinase [65], is known to negatively regulate the Wnt/β-catenin pathway [97], and cytoplasmic RARγ binds to and activates c-Src in a ligand-dependent manner [98].

To add to the role of RARγ in modulating survival versus cell death, its presence within the cytoplasm is required for the formation of the Ripoptosome (Ser/Thr kinases Receptor Interacting Kinase 1(RIPK1)/Receptor Interacting Kinase 3 (RIPK3)) death complex which drives necroptosis. Cisplatin and etoposide damage DNA, and the loss of RARγ within null mouse embryonic fibroblasts protected these cells against chemotherapeutic-induced necroptosis. These cells were also less susceptible to induction of the extrinsic apoptotic pathway by DNA-damaging agents. From studies of the induction of necroptosis, cytosolic RARγ initially formed a complex with RIPK1 but was not present in the final Riptosome complex containing RIPK3. From these findings, RARγ was proposed as a possible tumour suppressor because a significant decrease in expression was seen for squamous cell carcinoma biopsies [99]. Cytosolic RARγ has also been shown to play a role in tumour necrosis factor-induced cell death of HT-29 colorectal cancer cells when the cellular inhibitor of apoptosis (cIAP) activity was blocked [100].

The action of RARγ is most often considered in terms of the direct promotion of gene expression when ATRA is bound or suppression when the ligand is absent. Indeed, RARγ regulates the transcriptional activity of a very large gene network, as shown for F9 embryonal stem cells by integrative genomics. Post-treatment of F9 embryonal stem cells with ATRA, RARγ-retinoid X receptor α co-occupancy led to 281 genes exhibiting an induction of ≥1.8-fold by 2 h. Nine hundred and twenty-six genes were induced at 48 h [101]. Presently, we do not know how the antagonism of RARγ within, for example, prostate cancer cells effects gene expression. However, an interesting finding for gene expression relates to the translocation of RARγ from the nucleus to the cytoplasm and a reduction in gene occupancy and a lack of expression. This was seen when drug-resistant lung cancer-stem-cell-like cells were treated with the synthetic retinoid WYC-209 to drive apoptosis. The translocation of RARγ from the nucleus to the cytoplasm reduced the binding of RARγ to the cell division control protein 42 (Cdc42) promoter leading to down-regulated expression of Cdc42. Cdc42 activation regulates actin polymerisation, and reduced Cdc42 led to a decrease in filamentous actin, inhibition of cytoskeletal tension, and chromatin de-condensation [102]. Hence, RARγ plays a role in regulating the status of chromatin.

Blocking the action of RARγ by using an antagonist drives necroptosis. However, whether interference with the retinoic acid pathway by other means drives necroptosis and/or apoptosis isn’t entirely clear, and there is a need to resolve this matter. Moreover, RARγ appears to have multiple roles, with a role in the nucleus to regulate gene expression and in the cytoplasm to regulate signalling. To add to roles within the nucleus, RARγ plays a role in chromatin modelling because its presence is essential for ATRA-induced chromatin epigenetic marks and transcriptional activation within embryonic stem cells [103]. RAR/RXR dimers also play a role in the pluripotency of stem cells via their binding dynamics distinguishing pluripotency-associated from differentiation-associated cis-regulatory elements [27]. Regarding a role in pluripotency, RARγ’s action as an oncogene may be to disrupt chromatin modelling for pluripotency. A concept from findings for transgenic mice that developed leukaemias when the expression of various oncogenes was restricted to hematopoietic stem cells is that a feature of leukaemia stem cells is that their offspring are restricted to a cell lineage [104]. There is a need to further resolve the precise role of RARγ as an oncogene and its various roles in governing cell survival.

## 9. Are the Agents That Target the Retinoic Acid Pathway Selective for CSCs?

The agents that target RARγ have been shown to be more active against cancer cells than their normal tissue counterpart cells. Patients’ prostate cancer cells and human prostate cancer lines were more sensitive to the antagonism of RARγ and all RARs than normal human prostate epithelium and the human non-tumorigenic prostate line RWPE-1 [63,91]. Human fibroblasts express modest levels of RARα and RARβ [105], and normal human prostate fibroblasts were not affected by the pan-RAR antagonist [91]. As considered above, the enhanced sensitivity of patients’ prostate cancer cells seems to be due to the low level of ATRA within these cells. Acacetin was used to target RARγ within hepatocellular carcinoma, and there was strong inhibition of the growth of hepatocellular carcinoma cell lines and SW480 and SW620 colorectal cancer cell line cells. Normal LO2 liver cells were resistant to acacetin treatment [96].

Does antagonism of RARγ spare normal tissue-specific stem cells, which are essential to replenishing worn-out cells? The RARγ-selective and pan-RAR antagonists did not have any adverse effects when tested in vitro against purified human haematopoietic stem and progenitor cells (CD34+). The antagonism of RARγ did not have an observable effect on cultures [51]. The pan-RAR antagonist increased the culture lifespan from 20 days to up to 55 days, the number of stem (CD34+/CD133+) and progenitor cells, and the production of neutrophils and monocytes [51]. In keeping with the latter, a systemic expansion of myeloid cells was seen in mice that were made vitamin A deficient by dietary restriction [106]. It is interesting to note that in addition to killing CSCs, the pan-RAR antagonist is able to expand neutrophil production and may provide an adjunct to chemotherapy when life-threatening neutropenia is a side effect. The pan-RAR antagonist did not affect the viability and the cloning efficiency in micro-titre plate wells of the promyeloid cell line HL60, which expresses RARα but does not express RARγ [91]. Regarding mature blood cells, T lymphocytes express RARα and RARγ [107], and both antagonists did not affect the viability of purified peripheral blood lymphocytes when tested in vitro.

Direct information from in vitro studies of the effect of antagonising RARγ and all RARs on tissue-specific stem cells is still very limited largely because for tissues other than the hematopoietic cell system, it is difficult to distinguish stem cells with certainty from later progenitor cells. Even so, it is interesting to note that when mice were given daily doses of a substantial amount (5 mg/kg) of the pan-RAR antagonist BMS-18945 that there were no changes in body weight. Spermatogenesis was inhibited, but this was reversible. Other than in the testes, there were no detectable adverse effects in the organs examined, and the investigators have suggested the development of RAR antagonists as a male contraceptive [108]. In an earlier toxicological study, the testes of rats were also seen to be exquisitely sensitive to the effects of BMS-189453 with a dose of 10 mg/kg for 3 days producing no other signs of toxicity [109].

Silybin was used to target ALDH1 isoforms within prostate cancer cells, reduced the weight of tumour implants, and was not cytotoxic nor genotoxic at a concentration of 100 μM in animals. Silybin is the most active component within the extract from milk thistle seeds which is termed silymarin as to containing silybin A and B, isosilybin A and B, silibinin, silydianin, and silychristi. Silymarin was safe in humans at therapeutic doses, well tolerated at a high dose (700 mg three times a day for 24 weeks), and safe in pregnancy, as there were no anomalies in a clinical trial (reviewed in [110]). Solomargine was used to target ALDH1 isoforms within ovarian cancer cells and sensitised these cells to cisplatin and paclitaxel. In xenograft studies, the body weight of animals given a combined treatment was relatively unaltered, suggesting that solomargine has negligible toxicity [84].

## 10. The Prospect of Anti-CSC Agents

The future development of therapeutics that target the retinoic acid pathway to kill CSCs is highly promising. Historically, compounds from plants, including ones that have long been used in traditional folk medicine, have provided anticancer and other drugs. Silybin, from milk thistle seeds, and solomargine, from a species of nightshade, are ALDH inhibitors, and acacetin, from *Chrysanthemi indici,* targets RARγ. These compounds killed a range of cancer cells, spared normal tissue-counterpart cells, and reduced tumour growth in xenograft studies. Screening plant extracts for additional compounds that target the retinoic acid pathway and that are active against CSCs is highly likely to be a worthwhile endeavour. *Actinidia chinensi* planch root extract is a traditional Chinese medicine that inhibits the proliferation of a number of human cancer cell lines, and its action involves the RARβ-driven network [111]. A recent review has focused on glycoalkaloids from *Solanum* plants, like potato, aubergine, and tomato, because some of the compounds, like solamargine, are thought to kill CSCs [112].

The alternative approach to new drugs is medicinal chemistry which has delivered synthetic retinoids that are highly selective for RARγ; expression is restricted to primitive cells and antagonising drives necroptosis of CSCs. The enhanced sensitivity of prostate cancer CSCs as compared to normal prostate epithelial cells appears to relate to CSCs residing in a low ATRA sanctuary. This may also be the case for other carcinomas. There is a need for preclinical studies using animal models to examine whether antagonising RARγ prevents disease relapse and metastasis. Findings to date support the view that CSCs, or a subset of these cells, are responsible for metastasis, but the evidence is indirect [10]. Ascertaining whether antagonising RARγ prevents relapse and metastasis would address this issue and ensure that CSCs can be eradicated without long-term damage to normal tissue homeostasis. For all of the agents that target the retinoic acid pathway, there is a need for more detailed studies of their activity against a range of tissue-specific stem cells. Even so, the presence of quiescent CSCs confounds the use of the chemotherapeutics that are mostly used to treat cancer because they just kill dividing cells. The development of drugs that the target retinoic acid pathway may well provide a way forward to eradicating CSCs and a potential bona fide cure for aggressive and metastatic disease.

## 11. Concluding Remarks

The overexpression of RARγ and its activation play roles in the abnormal behaviour of CSCs for a number of cancers that so far include some cases of AML, cholangiocarcinoma, colorectal, clear cell renal cell, hepatocellular, pancreatic ductal adenocarcinoma, prostate, and ovarian. Tissue-specific stem cells are mostly at risk from transformation because their immortality guarantees that CSCs are immortal, as required to sustain cancer. There are many unanswered questions as to what extent and how malignancy might be a legacy of a perturbation to the ATRA control of the behaviour of a tissue-specific stem cell. CSCs and normal stem cells express RARγ for maintenance, and RARα, which is important to cell differentiation, and ATRA levels are tightly controlled by cells that synthesise and cells that degrade ATRA. RARγ is a conductor of the maintenance of normal stem cells and cell death of CSCs. To understand how the complex control of the local level of ATRA and the active or silent natures of RARγ and RARα underpin the development of normal stem cells and play a role in the malignant behaviour of CSCs, there is a need to unravel the extent to which these cells produce ATRA or such is made available by environmental cells. Regarding ATRA production, this might be difficult to measure, which is pertinent because RARγ is exquisitely sensitive to ATRA transactivation. ATRA is required for neural progenitors to arise from mouse embryonic stem cells, which produce ATRA endogenously, but this was not measurable. Instead, a developmental reliance was shown by either removing the all-*trans*-retinol that is needed to produce ATRA from the medium or the use of the pan-RAR antagonist AGN193109 to block the activity of RARs within the embryonic stem cells [113].

## Data Availability

Not applicable.
